# Enhancing efficiency and capacity of telehealth services with intelligent triage: a bidirectional LSTM neural network model employing character embedding

**DOI:** 10.1186/s12911-023-02367-1

**Published:** 2023-11-21

**Authors:** Jinming Shi, Ming Ye, Haotian Chen, Yaoen Lu, Zhongke Tan, Zhaohan Fan, Jie Zhao

**Affiliations:** 1 National Engineering Laboratory for Internet Medical Systems and Applications, The First Affiliated Hospital of Zhengzhou University, No.1 East Jianshe Road, Erqi District, Zhengzhou, Henan 450052 China; 2 National Telemedicine Center of China, The First Affiliated Hospital of Zhengzhou University, Zhengzhou, China

**Keywords:** Telehealth, Intelligent triage, Character embedding, Bidirectional LSTM

## Abstract

**Background:**

The widespread adoption of telehealth services necessitates accurate online department selection based on patient medical records, a task requiring significant medical knowledge. Incorrect triage results in considerable time wastage for both patients and medical professionals. To address this, we propose an intelligent triage model based on a Bidirectional Long Short-Term Memory (Bi-LSTM) neural network with character embedding to enhance the efficiency and capacity of telehealth services.

**Methods:**

We gathered a 1.3 GB medical dataset comprising 200,000 records, each including medical history, physical examination data, and other pertinent information found on the electronic medical record homepage. Following data preprocessing, a clinical corpus was established to train character embeddings with a medical context.

These character embeddings were then utilized to extract features from patient chief  complaints, and a 2-layer Bi-LSTM neural network was trained to categorize these complaints, enabling intelligent triage for telehealth services.

**Results:**

60,000 chief complaint-department data pairs were extracted from clinical corpus and divided into the training, validation, and test sets of 42,000, 9,000, and 9,000, respectively. The character embedding based Bi-LSTM neural network achieved a macro-precision of 85.50% and an F1 score of 85.45%.

**Conclusion:**

The telehealth triage model developed in this study demonstrates strong implementation outcomes and significantly improves the efficiency and capacity of telehealth services. Character embedding outperforms word embedding, and future work will incorporate additional features such as patient age and gender into the chief complaint feature to future enhance model performance.

## Background

Telehealth has emerged as a promising solution to mitigate the shortage of medical resources in various countries and regions and improve a nation’s overall healthcare level. This approach has been widely embraced in many countries across the world [1]. The Coronavirus Disease 2019(COVID-19) pandemic has further accelerated the adoption of telehealth technologies, such as telemedicine and teleconsultation, on a global scale [2, 3]. Telemedicine involves online communication between doctors and patients, while teleconsultation refers to professional consultation among doctors at different levels. Despite their increasing popularity, both telehealth services encounter a common challenge: how to accurately triage patients to the appropriate department based on their symptoms in an online setting. Therefore, the research question in this paper is how machine learning techniques can be leveraged to achieve intelligent triage for telehealth services.

During a visit to a physical hospital, there is usually a information desk to help patients choose the correct department. As telehealth service providers, large-scale hospitals usually offer a wide range of services and have a detailed classification of clinical departments. Choosing the correct department is often challenging for the initiators of telehealth services, whether telemedicine or teleconsultation mode. Telemedicine service initiators (usually referring to patients) lack professional medical knowledge. Nevertheless, in teleconsultation, primary health care professionals, as the initiator of services, do not know much about the department settings of higher-level medical institutions, so there may be the risk of the wrong choice of departments and doctors. Incorrect triage wastes the time of patients and experts and unnecessarily uses high-end telehealth equipment. These issues imply the necessity of an intelligent triage model during telehealth services. To address this practical issue, we have conducted research on the intelligent triage of telehealth.

The intelligent triage model for telehealth is designed to assist applicants in making informed decisions regarding the selection of the appropriate department for their remote medical activities. However, there is a lack of research specifically dedicated to intelligent guidance for telehealth. Current studies mainly concentrate on patient classification and limited prioritization within emergency departments [4–7], while the intelligent triage for telehealth focuses on the crucial task of accurately identifying the correct department. This particular challenge can be effectively solved as a text classification problem using machine learning [7]: how to classify the patient’s symptom text to the correct department. Several studies were carried out to predict the correct department according to symptoms. For example, using Term Frequency-Inverse Document Frequency (TF-IDF) and Support Vector Machine (SVM), Wang et al. trained and predicted 30,000 data from 13 departments, and the triage accuracy reached 76.3% [8]. Chen et al. conducted related research based on Multilayer Perceptron (MLP) and Long Short-Term Memory (LSTM) methods. In the text analysis of electronic medical records for a small amount of data, the deep learning intelligent diagnostic capabilities of the LSTM model cannot be fully utilized, and the MLP classification method in traditional machine learning has the best performance [9]. Dong et al. used the method of regular expression, based on International Classification of Diseases, Tenth Revision (ICD-10), to match patients’ initial diagnoses with the professional expertise of doctors in different departments and then realize intelligent triage [10]. Lu Liang et al. use the improved TF-IDF algorithm to calculate the weight of symptoms according to the user’s symptoms, making it more suitable for medical diagnosis [11]. However, most of the existing methods are based on word-level features, which may ignore some important information at the character level, especially for languages that do not have clear word boundaries, such as Chinese. We hypothesize that a model based on Bi-LSTM neural network with character embedding can effectively improve the accuracy and efficiency of telehealth triage.

To address the existing challenges in telehealth triage, we propose an intelligent triage model based on a Bi-LSTM neural network with character embedding. Specifically, we first extracted the chief complaints and treatment department data from electronic medical records, and formed a clinical corpus for training character embeddings with a medical background. Subsequently, these embeddings were used to extract features from patient chief complaints. Then, we trained a neural network containing a two-layer Bi-LSTM to predict the classification of the input complaints, resulting in the realization of intelligent triage for telehealth and a reduction in the workload of service providers. Our model can effectively improve the accuracy and efficiency of telehealth triage by extracting features from patient chief complaints at the character level, which is particularly significant for languages such as Chinese that lack clear word boundaries. Furthermore, compared to existing methods that rely on word-level features, our model is capable of capturing critical information from patient chief complaints and accurately routing them to the appropriate department.

The implementation of our model could have significant implications for the telehealth industry by improving the efficiency and capacity of telehealth services. By reducing the time required to triage patients and route them to the appropriate department, our model can increase the number of patients that can be served by telehealth services, which is especially important in areas with limited access to healthcare. Overall, our research represents a significant advancement in the field of telehealth triage and has the potential to improve healthcare outcomes for a wide range of patients.

## Methods

### Data set and feature selection

**Fig. 1:**
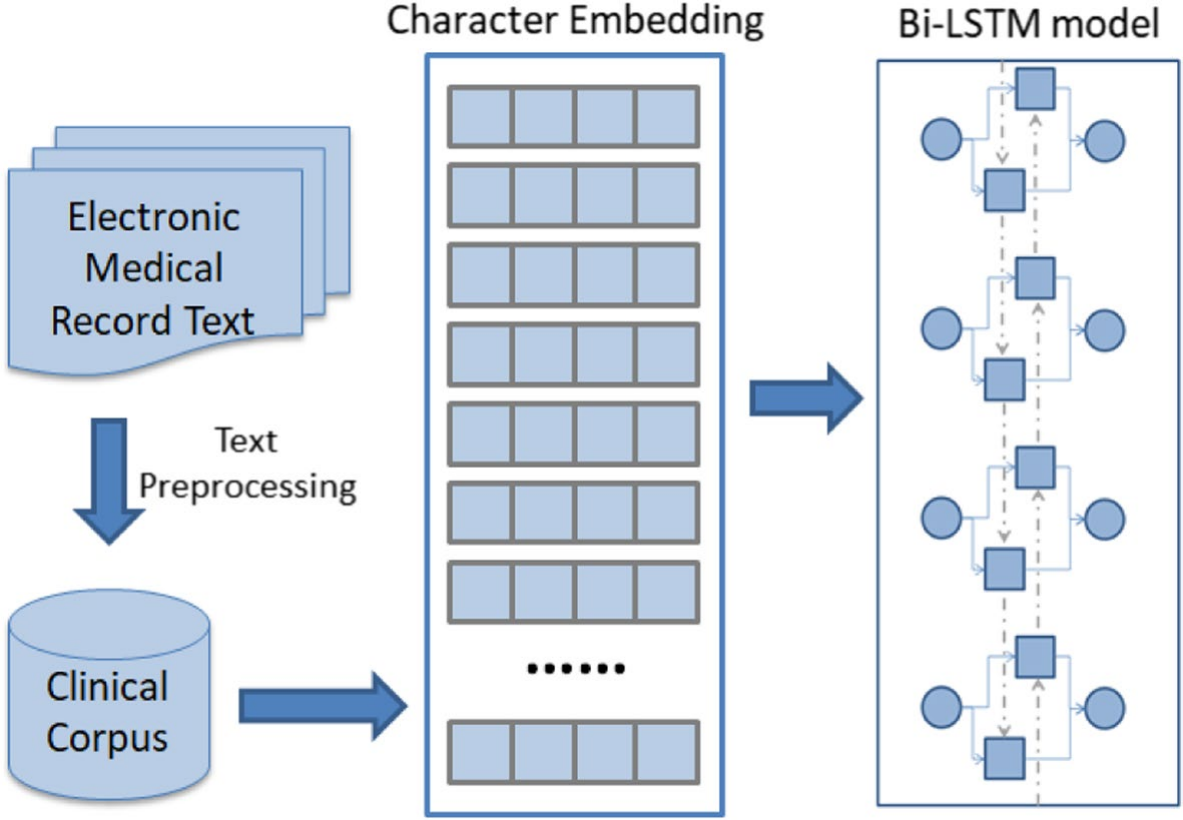
Structure diagram of intelligent triage

The model takes the patient’s chief complaint as input and outputs the corresponding department number. The flowchart of the method proposed in this study is shown in Fig. 1.

The electronic medical record text is preprocessed as raw data. During this process, useless information in the data is removed. This step mainly refers to removing stop words. Stop words are commonly used words in a language that carry little or no significant meaning in text analysis. In our study, we applied a stop word removal technique to eliminate these words from the electronic medical record text during the preprocessing stage. This step aims to reduce the impact of non-informative words on subsequent data processing and analysis. Examples of stop words include articles (e.g., “the”, “a”), prepositions (e.g., “in”, “on”), conjunctions (e.g., “and”, “but”), and other frequently occurring words that do not contribute substantially to the overall semantic understanding of the text. After preprocessing, the preprocessed data is formed into a clinical corpus to reduce the impact of useless information on embedding and improve the final performance.

Feature extraction is a crucial step following preprocessing in text analysis. In recent years, embedding has emerged as a prominent method for practical feature extraction in deep learning. Notable examples include Word2vec [12], GloVe [13], and others, which have been extensively utilized and validated [14, 15]. Compared to the traditional bag of words approach, embedding methods have demonstrated superior effectiveness. Word embedding, in particular, involves mapping words or phrases from the vocabulary to real-valued vectors, enabling the capture of semantic and syntactic similarity between words, thus providing useful information for text analysis [16, 17].

Another noteworthy approach is character embedding, which operates at the character level rather than the word level. In this method, each character is associated with a corresponding vector. Character embedding addresses certain challenges that word embedding struggles to tackle, such as out-of-vocabulary words, spelling errors, and abbreviations. Considering the unique characteristics of Chinese medical texts, this study argues that character embedding is better suited for extracting features in such contexts, consequently enhancing the overall performance of feature extraction.

To achieve this goal, we train character embedding using the preprocessed corpus with a size of 1.3 GB, which includes the homepage of electronic medical records and other related content. Due to the inherent differences between Chinese and English, Chinese text lacks natural word separators, necessitating the use of word segmentation techniques. However, medical word segmentation often yields suboptimal results. Such segmentation issues can lead to misleading interpretations, where character embedding sometimes performs better than word embedding [18, 19]. Therefore, this study proposes a novel approach based on character embeddings to capture word representations from the medical dataset, which serves as input to the LSTM network. By using this method based on character embedding, we can learn the embedding representation of each character from the medical dataset and use it as the input of a bidirectional LSTM neural network, thus improving the efficiency and capability of intelligent triage.

### Model development

A bidirectional LSTM neural network was developed in this study. LSTM is a variant of Recurrent Neural Network (RNN) equipped with forget, input, and output gates, is capable of selectively retaining or discarding information through these gates. This characteristic reduces the risk of gradient vanishing or explosion and enables the model to effectively capture long-distance dependencies [20]. The LSTM model has been extensively employed and explored in speech recognition and text classification tasks due to its effectiveness. Bidirectional LSTM can realize forward and reverse bidirectional encoding, better capturing bidirectional semantic dependencies [21].

**Fig. 2:**
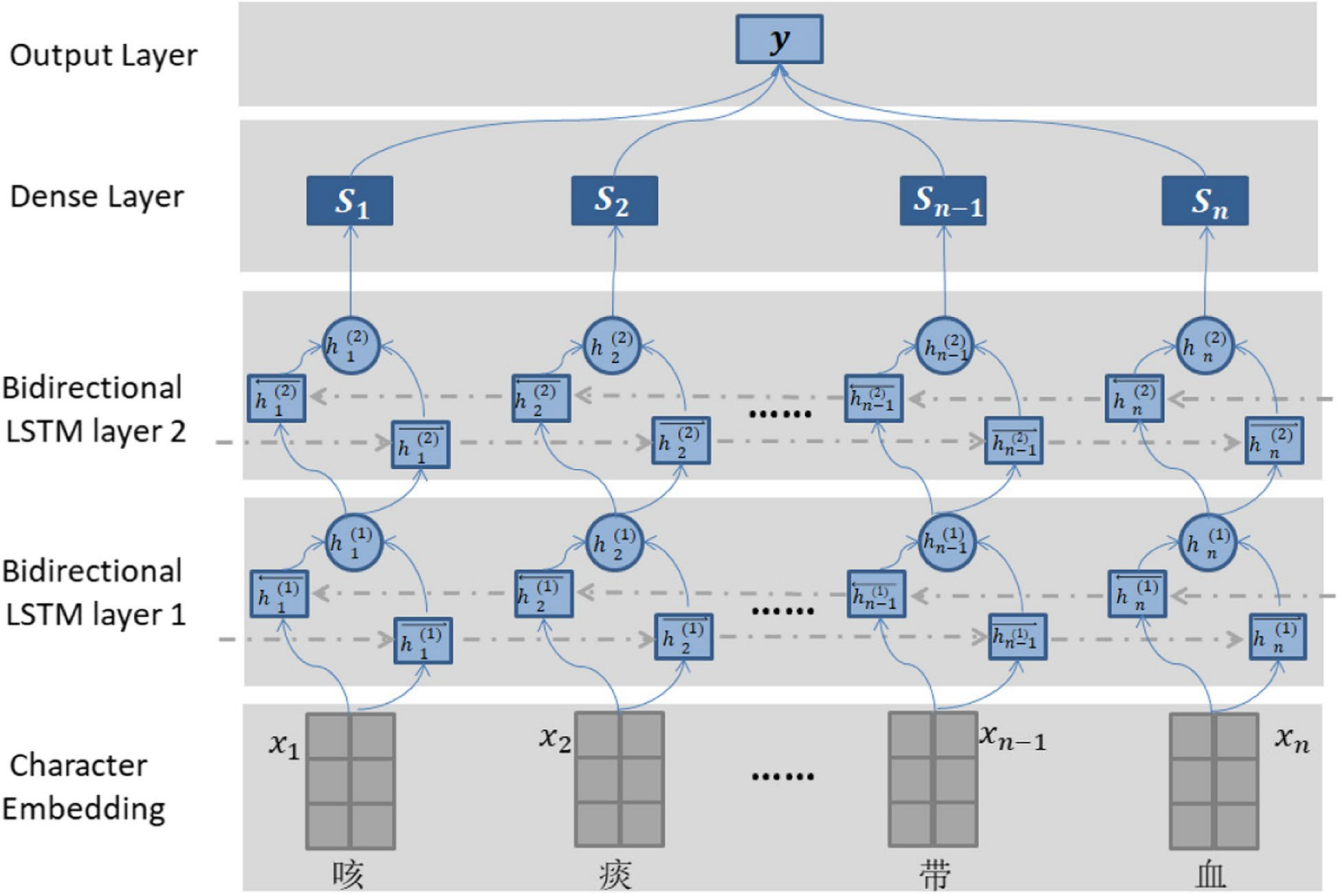
The Structure of the Proposed Intelligent Triage Model

The input of the network consists of feature vectors obtained from character embeddings based on the chief complaint text, while the output corresponds to the predicted classification result. In this study, a 2-layers Bi-LSTM neural network was proposed, it’s framework is illustrated in Fig. 2.

The network comprises five components, while the input is the feature vector of the chief complaint text, and the output corresponds to the department ID with the highest probability. As such, it can be regarded as a classification task in machine learning. The first component is the embedding layer, responsible for extracting the features from the chief complaint text, and “n” represents the length of the chief complaint text. Notably, the vector representation of each word is derived from a clinical corpus, trained with a medical background, rather than being randomly initialized. The feature vector of the chief complaint text serves as the input for the subsequent bidirectional LSTM layer.

The second and third components of the proposed architecture consist of two Bidirectional LSTM layers. Bidirectional LSTM is an enhanced version of LSTM RNN, has demonstrated superior performance across various domains, including speech recognition, natural language processing, sequence classification, and machine translation [21–23]. Unlike traditional LSTM, which processes information and extracts features in a single direction, Bidirectional LSTM overcomes this limitation. It concurrently trains both forward and backward LSTM networks, concatenating their outputs. This approach addresses the issue of unidirectional LSTMs being unable to encode information from the reverse direction.

**Fig. 3:**
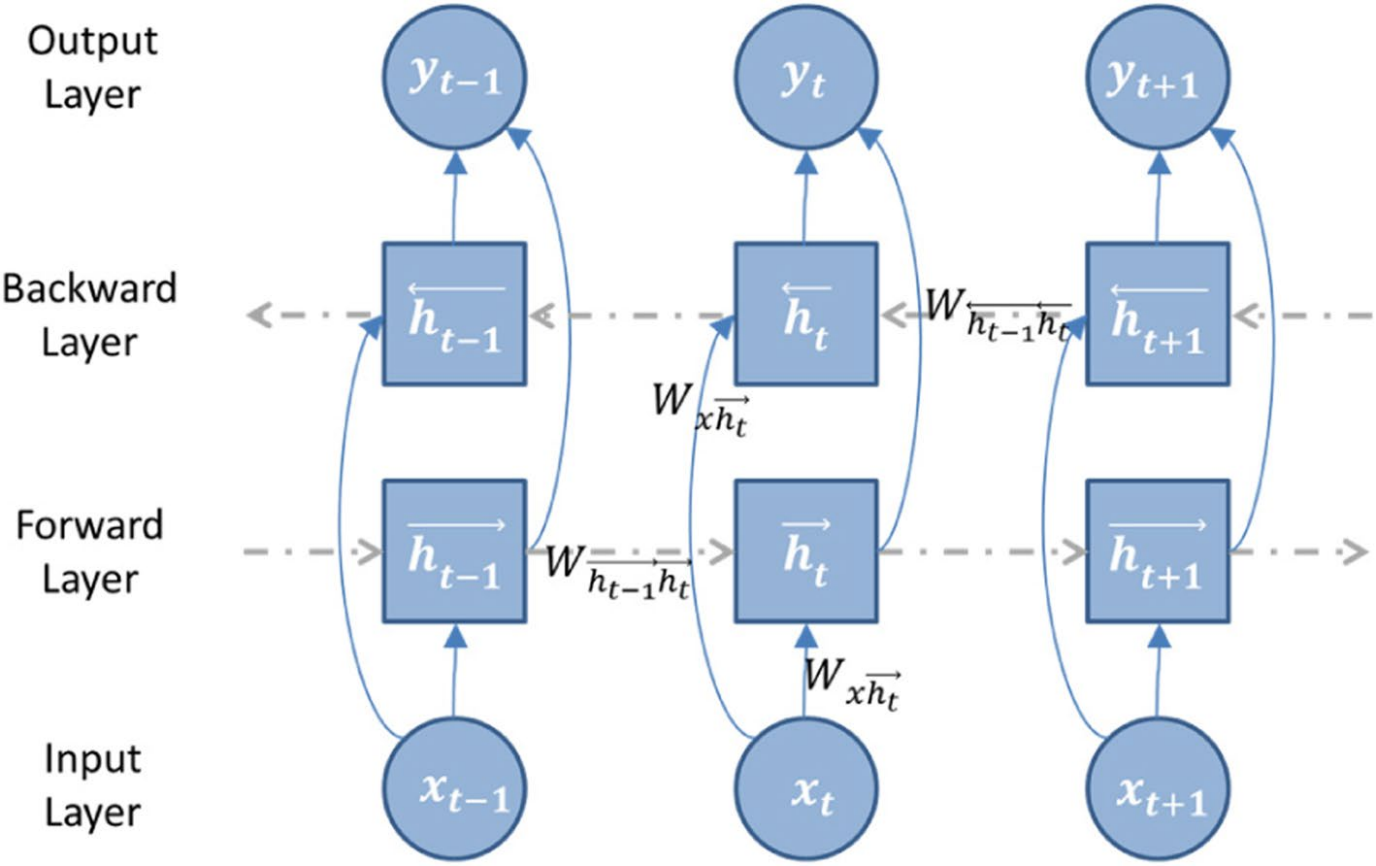
The architecture of the Bidirectional LSTM network

The neural network cell structure of Bidirectional LSTM is shown in Fig. 3.

Equation (1) shows the update formula for a forward recurrent neural network. The formula takes into account the previous hidden state $\overrightarrow{{h}_{t-1}}$, current input ${x}_{t}$, and bias term ${b}_{\overrightarrow{h}}$ to compute the new hidden state $\overrightarrow{{h}_{t}}$.

1 $$\overrightarrow{{h}_{t}}=H({W}_{x\overrightarrow{{h}_{t}}}{x}_{t}+{W}_{\overrightarrow{{h}_{t-1}}\overrightarrow{{h}_{t}}}\overrightarrow{{h}_{t-1}}+{b}_{\overrightarrow{h}})$$

Equation (2) shows the update formula for a backward recurrent neural network. The formula takes into account the previous hidden state$\overrightarrow{{h}_{t+1}}$, current input${x}_{t}$, and bias term ${b}_{\overleftarrow{h}}$ to compute the new hidden state$\overleftarrow{{h}_{t}}$.

2 $$\overleftarrow{{h}_{t}}=H({W}_{x\overrightarrow{{h}_{t}}}{x}_{t}+{W}_{\overleftarrow{{h}_{t-1}}\overleftarrow{{h}_{t}}}\overrightarrow{{h}_{t+1}}+{b}_{\overleftarrow{h}})$$

Equation (3) shows the formula for combining two neural networks. The formula takes into account the forward and backward recurrent neural network’s hidden states $\overrightarrow{{h}_{t}}$ and $\overleftarrow{{h}_{t}}$, and bias term ${b}_{{y}_{t}}$ to compute the new output ${y}_{t}$.

3 $${y}_{t}={W}_{\overrightarrow{{h}_{t}}{y}_{t}}\overrightarrow{{h}_{t}}+{W}_{\overleftarrow{{h}_{t}}{y}_{t}}\overleftarrow{{h}_{t}}+{b}_{{y}_{t}}$$

Finally, use the LSTM layer’s output as the dense layer’s input. The Dense layer is also called a fully connected layer. Its purpose is to perform the nonlinear transformation on the previously extracted features, extract the correlation between them, and then map them to the output. The formula for the fully connected layer is given by Eq. (4).

4 $$z=\sum\nolimits _{n=1}^{N}{W}_{n}{x}_{n}+b$$

Where *W* is the weight vector, *x* is the output of the Bidirectional LSTM layer, and *b* is the bias vector. The nonlinear function Logistic is selected as the activation function of the dense layer. Finally, a probability between [0, 1] is obtained.

## Results

### Experimental data

Relevant medical data has been obtained from the electronic health record system, and the data content contained a text-only medical data set of 200,000 records (about 1.3GB in size). A clinical corpus was constructed based on this data. In the corpus, we extracted 60,000 pairs of chief complaint-seeking department names from 20 departments, with 3000 pairs per department. The names of 20 departments are shown in Tables 1 and 2.

**Table 1:** List of 20 clinical departments

No.	Department Name	No.	Department Name
1	Breast Surgery	11	Gastroenterology
2	Obstetrics	12	Dermatology
3	Pediatrics	13	Ophthalmology
4	Endocrinology	14	Neurology
5	Stomatology	15	Neurosurgery
6	Respiratory Medicine	16	Otolaryngology
7	Otorhinolaryngology	17	Oncology
8	Gynecology	18	Hematology
9	Cardiology	19	Orthopedics
10	Urology	20	Rhinology

The data are divided into training, verification, and test sets. The training data sample size is 42,000, the verification data sample size is 9000, and the test data sample size is 9000.

**Table 2:** Experimental data explanation

	Each department data	Number of departments	Total
Training Set	2100	20	42,000
Validation Set	450	20	9000
Test Set	450	20	9000
Total	3000	20	60,000

### Evaluation setting

Our study was implemented on Tensorflow for python, and the kashgari tool [24] was used to implement Bi-LSTM neural network construction quickly. Table 3 shows the hyperparameters set used in our study.

**Table 3:** The hyperparameters set

Parameter	Value
Character embedding size	100
Character embedding window	2
Character embedding min_count	20
Training epoch	10
Batch size	64
1st layer LSTM units	128
2nd layer LSTM units	64
Dropout rate	0.5

### Evaluation metrics

To evaluate the performance of intelligent triage, Precision (P), Recall (R), and F1-score were used. The relevant formulas are as follows:

5 $$P=\frac{TP}{TP+FP}$$

6 $$R=\frac{TP}{TP+FN}$$

7 $$F1=\frac{2*P*R}{P+R}$$

8 $$macro-P=\frac{1}{n}\sum\nolimits _{i=1}^{n}{P}_{i}$$

9 $$macro-R=\frac{1}{n}\sum\nolimits _{i=1}^{n}{R}_{i}$$

10 $$macro-F1=\frac{2*{P}_{macro}*{R}_{macro}}{{P}_{macro}+{R}_{macro}}$$

TP stands for True Positive, FP stands for False Positive, and FN stands for False Negative. Macro-Precision, Macro-Recall, and Macro-F1-score are used to evaluate the overall performance of the system.

### Analysis results

In order to verify the effectiveness of the Bi-LSTM network in telehealth triage, the single-layer Bi-LSTM model and CNN_Attention model were selected for comparison. We also explore the effect of character embedding and provide results under different hyperparameters.

### Performance comparison of the Bi-LSTM model and basic model

Single-layer Bi-LSTM and CNN-Attention are selected as baselines to evaluate the effectiveness proposed model. The performance of each model on the same dataset is presented in Table 4. The results demonstrate that the double-layer Bi-LSTM has a 0.43% improvement in macro-F1 compared to the single-layer one. This indicates that adding an additional Bi-LSTM layer enables the neural network to learn more features by utilizing a greater number of parameters.

The CNN_Attention model is widely researched and applied in text classification. On the same dataset, CNN_Attention achieves acceptable performance with a macro-F1 of 84.66%. Our proposed model surpasses both the single-layer bidirectional LSTM and the popular CNN-Attention model, with macro-F1 improvements of 1.89% and 0.79%, respectively. Compared to the two-layer Bi-LSTM model, our model also demonstrates an improvement of 1.47% in macro-F1. These improvements can be attributed to character embedding trained in the context of the clinical corpus, emphasizing the significance of incorporating medical background knowledge for department classification.

**Table 4:** Performance comparison of different models

*Model*	*macro-P*	*macro-R*	*macro-F1*
Bi-LSTM	83.97%	83.67%	83.56%
CNN_Attention	84.80%	84.75%	84.66%
2 layers-Bi-LSTM	84.40%	84.08%	83.98%
2 layers-Bi-LSTM + character embedding	85.50%	85.55%	85.45%

When using the set of hyperparameters listed in Table 3, the model achieves the best results with macro-P of 85.50%, macro-R of 85.55%, and macro-F1 of 85.45%. The prediction results for each department under these conditions are illustrated in the following figure. The macro-P in Dermatology was the highest at 98.49%, the highest macro-R in ophthalmology was 99.54%, and the highest macro-F1 in ophthalmology was 98.75%. Among all 20 departments, the macro-F1 value exceeds 85% in 10 departments, while the model performs relatively poorer in the remaining ten departments. This discrepancy is attributed to the less distinctive characteristics of chief complaints in departments such as oncology and neurosurgery. In actual clinical practice, patients are referred to the appropriate department after an initial definitive diagnosis by physicians with the aid of relevant tests. Some cases may require visits to multiple relevant departments (Table 5).

**Table 5:** Performance comparison of each department

	*Department Name*	*macro-P*	*macro-R*	*macro-F1*
1	Breast Surgery	98.00%	98.88%	98.44%
2	Obstetrics	95.77%	91.07%	93.36%
3	Pediatrics	79.22%	78.21%	78.71%
4	Endocrinology	90.66%	86.15%	88.35%
5	Stomatology	94.70%	89.94%	92.26%
6	Respiratory Medicine	72.64%	72.64%	72.64%
7	Otorhinolaryngology	80.04%	80.21%	80.13%
8	Gynecology	86.60%	91.46%	88.96%
9	Cardiology	83.37%	85.93%	84.63%
10	Urology	86.47%	95.04%	90.55%
11	Gastroenterology	80.13%	84.55%	82.28%
12	Dermatology	**98.49%**	97.44%	97.96%
13	Ophthalmology	97.96%	**99.54%**	**98.75%**
14	Neurology	77.85%	75.61%	76.72%
15	Neurosurgery	69.71%	78.32%	73.77%
16	Otolaryngology	94.95%	96.64%	95.79%
17	Oncology	71.04%	57.78%	63.73%
18	Hematology	82.08%	80.52%	81.29%
19	Orthopedics	88.91%	91.34%	90.11%
20	Rhinology	81.47%	79.69%	80.57%

### Performance comparison of character embedding and word embedding

Character embedding, constructed based on the clinical corpus, encompasses a total of 3865 character embeddings, which contributes significantly to the improved performance of our model. To demonstrate this, we trained character embedding using a news corpus under identical parameters, datasets, and model settings. Additionally, we trained a word embedding model on the clinical corpus for comparison purposes. The comparison results are presented in Table 6.

**Table 6:** Performance comparison with different embedding

*Model*	*macro-P*	*macro-R*	*macro-F1*
2 layers-Bi-LSTM + word embedding(news corpus)	83.36%	83.21%	83.11%
2 layers-Bi-LSTM + word embedding(clinical corpus)	84.55%	84.53%	84.49%
2 layers-Bi-LSTM + character embedding(clinical corpus)	85.50%	85.55%	85.45%

The data reveals that word embedding trained on the clinical corpus outperforms those trained on the news corpus, resulting in a 1.18% increase in the macro-F1 value. These results demonstrate the contribution of medical background knowledge to telehealth triage. Furthermore, character embedding exhibits improved performance compared to word embedding, with a macro-F1 value increase of 2.34%. This reinforces the notion that training and implementing character embedding effectively mitigate performance degradation caused by errors in medical text segmentation.

### Performance comparison of different hyperparameters

**Fig. 4:**
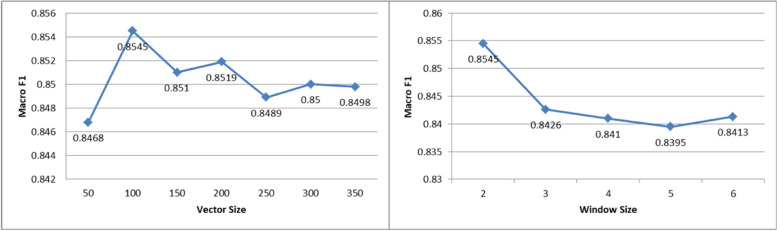
Macro F1 values under different vector sizes and window sizes

Hyperparameters play a crucial role in the character embedding and neural network, with significant variations across different parameters. Among these parameters, vector size and window size are particularly critical. The vector size represents the dimensionality of the embedding vectors, while the window size refers to the maximum distance between the current and predicted word within a sentence. Figure 4 illustrates the performance comparison of different vector and window sizes.

The results indicate that the highest macro F1 score of 85.45% is achieved when the vector size is set to 100 and the window size is set to 2. Additionally, it is worth noting that while word units are commonly used in training machine learning models, Chinese text lacks natural delimiters between words. Consequently, segmenting the chief complaint text before training the paragraph vectors becomes necessary. However, due to the short length and presence of specialized terms in the chief complaint text, word segmentation does not yield optimal results, thereby affecting the model’s performance.

To analyze the difference in the model’s performance under different parameters, the following explanations are provided:

- When the vector size is set to 100, the character embedding adequately captures the required semantic information for model. Setting the dimension too high resulting in sparse data, while setting it too low leads to the loss of certain semantic information.
- The model performs best when the window size is set to 2. This is because chief complaint texts are typically short. If the window size is too large, it results in a high number of training data. A window size of 2 provides the optimal effect. Conversely, a window size of 1 fails to capture the necessary semantic information with paragraph vectors.

## Discussion

Accurate and timely triage significantly influences the efficiency and quality of telehealth services. This study aims to address the limitations of manual triage in telehealth, and develops an intelligent triage model that uses a Bi-LSTM neural network and character embedding to improve telehealth’s the efficiency and capacity. In this section, we will discuss our model’s advantages, contributions, comparisons, challenges, and limitations.

Our novel approach of using character embedding avoids classification errors caused by medical text segmentation, improving the model’s accuracy and robustness. Character embedding is a technique that represents each character in a text as a vector of numerical values, capturing the semantic and syntactic information of the character. By using character embedding, we can avoid the problem of word segmentation in Chinese medical texts, which is often ambiguous and error-prone. Simultaneously, by employing a Bi-LSTM neural network, we harness the powerful expressive capacity and long-term memory mechanisms of neural networks, enabling better capture of semantic information and contextual relationships in patients’ symptom descriptions. Bi-LSTM neural network is a type of recurrent neural network that can process sequential data from both forward and backward directions, enhancing the model’s ability to learn from long-term dependencies.

We also reviewed some related works that have attempted to solve the same or similar problems as ours, and compared our intelligent triage model with other existing methods, including expert systems [25–28], similarity calculation methods [29, 30], and more traditional machine learning algorithms. Liefold achieves service grading and higher-level treatment decisions by evaluating past medical history and current symptoms [31]. Wang et al. also treated this task as a multi-classification problem, and classified 33,073 data from 13 departments using the TF-IDF vectorization method. They chose the support vector machine as the model and achieved a result of 76.3% [32]. Abdaoui et al. compared five classification algorithms: SVM, naive Bayes, random forest, decision tree J48 and JRip, and built a binary classifier for each category. Finally, they recommended suitable doctors for patients [33]. Diao et al. transformed this task into a three-layer classification problem, following the hierarchical order of department-secondary department-major disease, and classified the patient’s questions layer by layer. Then they used a clustering-based collaborative filtering recommendation algorithm to complete the recommendation of departments and doctors [34]. Our model outperforms these techniques, achieving a macro-average F1 score of 85.45% using only symptom descriptions as input. The result showed that our method has the ability to overcome the shortcomings of these systems, such as the excessive complexity and high cost of knowledge combination and knowledge base maintenance in expert systems, and the inability to cope with multiple coexisting diseases in similarity calculation methods.

While our model demonstrates promising results in intelligent triage for telehealth services, there are still limitations and areas for improvement in this research. We solely utilized symptom descriptions as input features, without considering other potentially influential patient-related information such as age, gender, and medical history. Future research could explore including additional features and apply feature selection algorithms to identify the most informative features to improve classification performance. The dataset used in our study is relatively small, potentially impacting the model’s generalizability. Future work could consider expanding the dataset and compare the model’s performance across different dataset sizes. Additionally, our model was developed specifically for telehealth services in China. It may not fully address the characteristics and demands of remote healthcare services in other countries or regions. Future research could explore adapting this model to different languages, cultures, and healthcare systems to enhance its universality and practicality. Notably, the current evaluation of our Bi-LSTM model is predominantly on its learning performance, using metrics like macro-accuracy and F1 scores. We acknowledge this might not directly tackle the real-world efficacy of departmental recommendations in telehealth contexts, especially regarding patient satisfaction and the precision and accuracy of recommendations when physicians are involved. In subsequent studies, we plan to incorporate supplementary evaluation measures that stress the model’s tangible implications in real scenarios, ensuring that patient outcomes and physician feedback remain paramount.

## Conclusions

This study presents an intelligent triage model based on a Bi-LSTM network to address the limitations of manual triage in telehealth services. By employing character embedding and a two-layer Bi-LSTM neural network, we achieve efficient and accurate triage, thus partially improving the efficiency and capacity of telehealth services.

Our intelligent triage model offers several significant scientific contributions. Firstly, by utilizing character embedding, we address the challenge of classification errors caused by medical text segmentation. This technique represents each character in the text as a numerical vector, capturing both semantic and syntactic information. Consequently, our model avoids the ambiguity and errors associated with word segmentation, particularly in Chinese medical texts.

Secondly, the incorporation of a bidirectional LSTM neural network enables our model to harness the expressive capacity and long-term memory mechanisms of neural networks. By processing sequential data from both forward and backward directions, our model excels at capturing semantic information and contextual relationships within patients’ symptom descriptions. This ability enhances the model’s capacity to learn from long-term dependencies and make accurate triage decisions.

Moreover, our intelligent triage model contributes to the field by enhancing the efficiency and quality of telehealth services. With an impressive macro-average F1 score of 85.45%, the model outperforms other techniques based on an expert system or similarity calculations. By relying solely on symptom descriptions as input, our model guides patients to suitable departments, streamlining the triage process and ensuring timely access to appropriate healthcare services.

In summary, our model’s primary scientific contributions lie in its effective use of character embedding to overcome classification errors caused by text segmentation, as well as its utilization of a bidirectional LSTM neural network to capture semantic information and contextual relationships. These contributions substantially enhance the efficiency and quality of telehealth services. These research findings offer novel avenues for triage systems in telehealth, enabling patients to benefit from more convenient and personalized medical services.

## Data Availability

The datasets generated and/or analyzed during the current study are not publicly available due to the confidentiality policy of the first affiliated hospital of Zhengzhou university but are available from the corresponding author on reasonable request and with permission of the first affiliated hospital of Zhengzhou university.

## Abbreviations

COVID-19: Coronavirus Disease 2019
TF-IDF: Term Frequency-Inverse Document Frequency
SVM: Support Vector Machine
MLP: Multilayer Perceptron
LSTM: Long Short-Term Memory
ICD10: International Classification of Diseases, Tenth Revision
Bi-LSTM: Bidirectional Long Short-Term Memory
RNN: Recurrent Neural Network
CNN_Attention: Convolutional Neural Network with Attention
J48: C4.5 Decision Tree Classifier
JRip: RIPPER Decision Tree Classifier
Word2vec: Word to Vector
GloVe: Global Vectors for Word Representation
